# Expert Consensus Document: Diagnosis for Chronic Constipation with Faecal Retention in the Rectum Using Ultrasonography

**DOI:** 10.3390/diagnostics12020300

**Published:** 2022-01-25

**Authors:** Masaru Matsumoto, Noboru Misawa, Momoko Tsuda, Noriaki Manabe, Takaomi Kessoku, Nao Tamai, Atsuo Kawamoto, Junko Sugama, Hideko Tanaka, Mototsugu Kato, Ken Haruma, Hiromi Sanada, Atsushi Nakajima

**Affiliations:** 1 School of Nursing, Ishikawa Prefectural Nursing University, 1-1 Gakuendai, Kahoku 929-1210, Japan; matumoto@ishikawa-nu.ac.jp; 2 Department of Gerontological Nursing/Wound Care Management, Graduate School of Medicine, The University of Tokyo, 7-3-1 Hongo, Bunkyo-ku, Tokyo 113-0033, Japan; hsanada@g.ecc.u-tokyo.ac.jp; 3 Department of Gastroenterology and Hepatology, School of Medicine, Yokohama City University, 3-9 Fukuura, Kanazawa-ku, Yokohama 236-0004, Japan; nobomisa@yokohama-cu.ac.jp (N.M.); takaomik@yokohama-cu.ac.jp (T.K.); 4 Department of Gastroenterology, National Hospital Organization Hakodate National Hospital, 18-16 Kawahara-cho, Hakodate 041-8512, Japan; momoko0221tsuda@gmail.com (M.T.); mkato1957@gmail.com (M.K.); 5 Division of Endoscopy and Ultrasonography, Department of Clinical Pathology and Laboratory Medicine, Kawasaki Medical School, 2-6-1 Nakasange, Kita-ku, Okayama 700-8505, Japan; n_manabe@med.kawasaki-m.ac.jp (N.M.); kharuma@med.kawasaki-m.ac.jp (K.H.); 6 Department of Palliative Medicine, Yokohama City University Hospital, 3-9 Fukuura, Kanazawa-ku, Yokohama 236-0004, Japan; 7 Department of Imaging Nursing Science, Graduate School of Medicine, The University of Tokyo, 7-3-1 Hongo, Bunkyo-ku, Tokyo 113-0033, Japan; ntamai@m.u-tokyo.ac.jp; 8 Global Nursing Research Center, Graduate School of Medicine, The University of Tokyo, 7-3-1 Hongo, Bunkyo-ku, Tokyo 113-0033, Japan; 9 Division of Ultrasound and Department of Diagnostic Imaging, Tokyo Medical University Hospital, 6-7-1 Nishishinjuku, Shinjuku-ku, Tokyo 160-0023, Japan; a-kawamo@tokyo-med.ac.jp; 10 Research Center for Implementation Nursing Science Initiative, School of Health Sciences, Fujita Health University, 1-98 Dengakugakubo, Kutsukake-cho, Toyoake 470-1192, Japan; junko.sugama@fujita-hu.ac.jp; 11 School of Nursing, College of Nursing and Nutrition, Shukutoku University, 673 Nitona-cho, Chuo-ku, Chiba City 260-8703, Japan; hitanaka@soc.shukutoku.ac.jp

**Keywords:** constipation, faecal retention, rectum, ultrasonography

## Abstract

Chronic constipation is a common gastrointestinal disorder in older adults, and it is very important to manage chronic constipation. However, evaluating these subjective symptoms is extremely difficult in cases where patients are unable to express their symptoms because of a cognitive or physical impairment. Hence, it is necessary to observe the patient’s colonic faecal retention using objective methods. Ultrasonography observation for colonic faecal retention is useful for diagnosing constipation and evaluating the effectiveness of treatment. Since there was no standard protocol for interpreting rectal ultrasonography findings, we developed an observation protocol through an expert consensus. We convened a group of experts in the diagnosis and evaluation of chronic constipation and ultrasonography to discuss and review the current literature on this matter. Together, they composed a succinct, evidence-based observation protocol for rectal faecal retention using ultrasonography. We created an observation protocol to enhance the quality and accuracy of diagnosis of chronic constipation, especially rectal constipation. This consensus statement is intended to serve as a guide for physicians, laboratory technicians and nurses who do not specialise in ultrasound or the diagnosis of chronic constipation.

## 1. Introduction

Chronic constipation is a common gastrointestinal disorder in older adults. Chronic constipation occurs in 16% of adults, with higher rates in older patients [1]. About one-third of adults aged 60 years or older report at least occasional constipation [1], and the prevalence is 50% or more in nursing home residents [2]. Moreover, chronic constipation is more commonly observed in women and those with lower socioeconomic status [3,4]. Recently, it has been shown that the prognosis of constipated patients is significantly worse than that of patients without constipation [5]. Constipated patients have an increased risk of cardiovascular events, possibly due in part to cardiovascular stress caused by anger during defaecation. Epidemiological studies in Japan have reported a significant increase in cardiovascular events, as the frequency of defaecation decreases [6]. Therefore, the management of chronic constipation is very important.

The Rome IV criteria categorise disorders of chronic constipation into four subtypes: (a) functional constipation; (b) irritable bowel syndrome with constipation; (c) opioid-induced constipation; and (d) functional defaecation disorders, including inadequate defaecatory propulsion and dyssynergic defaecation [7]. Functional constipation is the most common type of chronic constipation and is generally diagnosed based on symptoms according to the Rome IV diagnostic criteria [1]. To define functional constipation, ≥2 of the following symptoms should be present: straining at stool, lumpy or hard stool, sensation of incomplete evacuation, sensation of anorectal obstruction or blockage, need for manual manoeuvres and <3 bowel movements per week. However, three of the six Rome IV items require subjective evaluation, which is extremely difficult if patients are unable to communicate their symptoms because of a cognitive or physical impairment. Hence, it is necessary to observe patients’ colonic faecal retention using objective methods.

Diagnostic tests are typically recommended for constipation to evaluate the rectum and colon include plain abdominal X-ray (radiography), barium enema, colonoscopy, defaecography, abdominal computed tomography and magnetic resonance imaging [8,9,10]. However, these procedures involve disadvantages such as being invasive with radiation exposure, requiring long examination times or potentially providing inadequate information; moreover, they are unsuitable for follow-up testing, are expensive and lack standardisation. X-rays are a test that can be performed in many hospitals, but the cost is moderate, and the information is often unclear. Conversely, conventional ultrasonography (US) can be broadly applied in clinical practice due to its advantages of low cost, high safety, high speeds and use of non-invasive, nonionising radiation [11,12]. Recently, there has been a dramatic increase in the use of point-of-care ultrasound (POCUS) using a handheld ultrasound device by physicians or nurses who do not specialise in USs. Such handheld devices enable physicians or nurses to observe the subject at the bedside and make on-the-spot decisions for care. Ideally, POCUS can be used by anyone in a multidisciplinary team to observe faecal retention for the evaluation of constipation.

The US observation of colonic faecal retention is useful for diagnosing constipation and evaluating the effectiveness of treatment. Manabe et al. [13,14] reported that the responsiveness of patients with chronic constipation to medical treatment may be reflected in two parameters (the constipation index and the left/right distribution ratio) used to evaluate patients’ stool and/or gas distribution, which are calculated by the US observation of the colon. Thus, to properly assess constipation, the entire colon should be evaluated with US; however, not everyone in a multidisciplinary team has the high level of skill required for this type of assessment. The literature has asserted the importance of observing the presence or absence of rectal faecal retention when assessing constipation in older adults [15]; additionally, educational programmes have been developed for nurses [16], and the effectiveness of rectal US-based defaecation care in a home-care setting has been verified [17]. To build on this prior work, we decided to focus on the use of rectal US to confirm faecal retention.

A hyperechoic area shaped like a half-moon on a US reflects faecal retention in the rectum. Furthermore, an acoustic shadow on the crescent-shaped hyperechoic area in the US reflects hard stool retention. Tanaka et al. reported that 100% of older adult patients with a half-moon shape in the rectal hyperechoic area had a decreased frequency of defaecation and 92.9% of older adult patients with an acoustic shadow on the crescent-shaped hyperechoic area had hard stools. Several previous studies in children also support the use of rectal US in diagnosing constipation [18,19,20]. However, until now, there has not been a standard protocol for observing constipation via rectal US; therefore, we sought to develop an observation protocol through expert consensus.

## 2. Methods

Our aim was to create an observation protocol to enhance the quality and accuracy of diagnoses of chronic constipation, especially rectal constipation. This consensus statement is intended to serve as a guide for physicians, laboratory technicians and nurses who do not specialise in US or the diagnosis of chronic constipation. For a narrative review of the current literature in this area, we convened a group of gastroenterologists, sonographers, nurses and a wound, ostomy and continence nurse (WOCN) with expertise in the diagnosis and evaluation of chronic constipation and US. The criteria for the selection of the members were as follows: (1) research achievements on the evaluation of constipation via US in Japan; or (2) being the president of an academic society with high expertise in the diagnosis, assessment, treatment and defaecation care of constipation. From July to October 2020, online working group meetings were conducted once a month (a total of four meetings) to create the content of the consensus. Subsequently, another meeting was conducted for the entire membership to reach a consensus. Following discussion and agreement, they composed a succinct, evidence-based observation protocol for rectal faecal retention using US. The content of this consensus document was presented at the 22th meeting of the Japanese Society of Neurogastroenterology in Tokyo, Japan, on 20 November 2020, and a final version was developed after further discussion and agreement.

## 3. Protocol for the Observation of Rectal Faecal Impaction Using US

### 3.1. Subjects

The target population for this US observational protocol is individuals with suspected constipation. Subjects who can be interviewed are evaluated for suspected constipation according to the Rome IV diagnostic criteria [7]. For subjects who cannot be interviewed, it is necessary to obtain other objective information. For example, the Bristol Stool Form Score should be used to assess faecal characteristics, and constipation should be suspected when 1–2 hard stools are observed [21]. Constipation should also be suspected, when the stool volume is clearly low in relation to food intake.

### 3.2. Timing of the Observation

It is preferable to perform US observation, when there is urine retention in the bladder (since the bladder can be used as an acoustic window), when the patient does not eat immediately prior to the exam (since there is no effect of small bowel contents or gas) and when there is no gas retention in the abdomen.

### 3.3. Observation Procedure

#### 3.3.1. Position/Posture

The patient should be supine and should be comfortable. The head should be propped at about 10 degrees, with a bath towel or cushion placed under the neck to relieve tension in the abdomen, and the knee joints should be gently flexed.

#### 3.3.2. US Devices

A convex probe should be selected for the US, preferably with a bandwidth of 2–5 MHz in frequency. The required resolution is based on the following two conditions: (a) the bladder can be depicted as an anechoic area with a few multiple reflections; and (b) the boundaries and edges of the tissue can be depicted uniformly (i.e., the boundaries between the rectal contents and the intestinal tract and the bladder wall can be clearly depicted). 

In addition to the US device, the procedure requires echo jelly, hand towels and tissue paper for wiping off the jelly and the blanket.

#### 3.3.3. Flowchart of the Rectal US Observation

**Figure 1:**
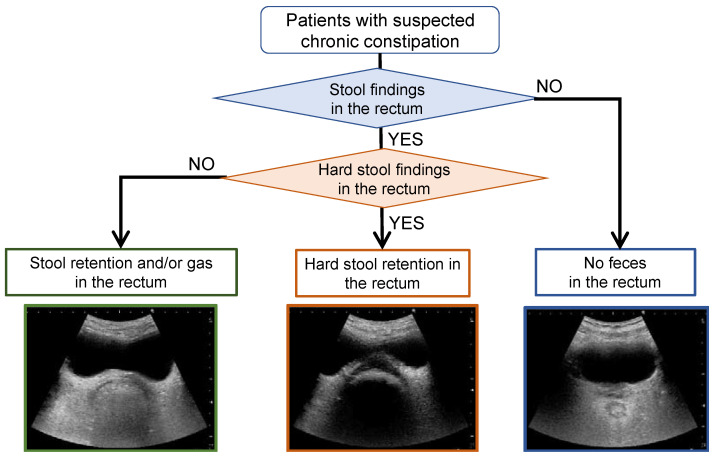
Flowchart for the observation of rectal faecal retention with ultrasonography.

Figure 1 presents a flowchart of the rectal US observation to evaluate faecal retention. This flowchart is intended for use in individuals with suspected constipation, where US is used to confirm the presence, properties, location and volume of rectal faecal retention and to select treatment and care. In this flowchart, transverse US images confirm the presence and nature of rectal faecal retention, and longitudinal US images confirm the location and amount of rectal faecal retention. Here, rectal gas was classified in the same group as rectal faecal retention, because it is sometimes difficult to distinguish rectal gas from faecal retention on US images and because the diagnosis is not significantly affected even when faecal retention cannot be distinguished from rectal gas.

#### 3.3.4. Probe Scanning and Typical Images

**Figure 2:**
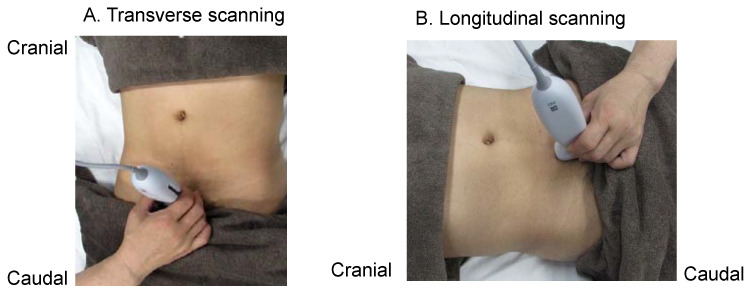
How to apply the ultrasound probe. The probe is placed at the superior margin of the pubis for both transverse scanning (**A**) and longitudinal scanning (**B**). In both techniques, the ultrasound beam is tilted caudally by 10–30 degrees to visualise the bladder. The bladder is used as an acoustic window, with the rectum being visualised deeper than the bladder.

In both transverse and longitudinal scans, the US probe is placed at the superior border of the pubic bone, and the US beam is tilted caudally 10–30 degrees to visualise the bladder (Figure 2). The bladder is used as an acoustic window, and the rectum visualisation is deeper than that of the bladder. 

**Figure 3:**
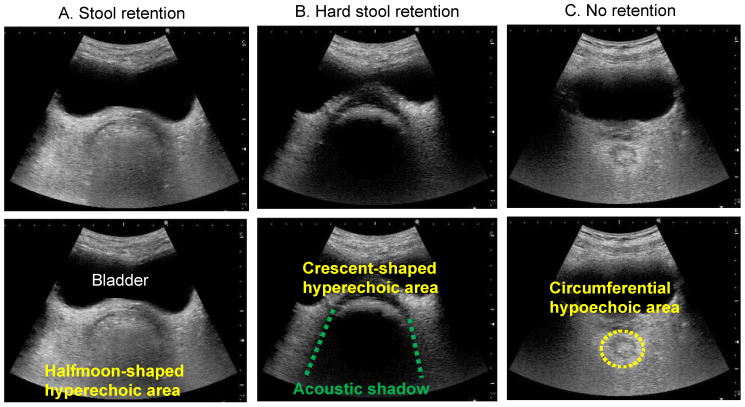
Transverse rectal ultrasound images showing the presence of stool and hard stool. The top three images are the original ultrasound images, and the bottom three images illustrate the ultrasound findings. (**A**) Stool retention. A halfmoon-shaped hyperechoic area is observed in the lower part of the bladder. (**B**) Hard stool retention. A crescent-shaped hyperechoic area with an acoustic shadow is observed in the lower part of the bladder. (**C**) No retention. No hyperechoic area is observed, because there is no faecal retention. A circumferential hypoechoic area is observed in the lower part of the bladder.

When there are contents (faecal retention) in the rectal lumen, US waves are reflected from the surface of the contents at a depth deeper than the bladder (anechoic area) and a hyperechoic area with a half-moon shape is depicted in the transverse US image (Figure 3A). In addition, when there is hard stool accumulation, an acoustic shadow is depicted in the transverse US image of the crescent-shaped hyperechoic area (Figure 3B). In contrast, if there is no faecal retention or gas in the rectal lumen, no obvious hyperechoic area is depicted. In the transverse US image, a circumferential hypoechoic area may be observed as an empty intestine (Figure 3C) [22].

**Figure 4:**
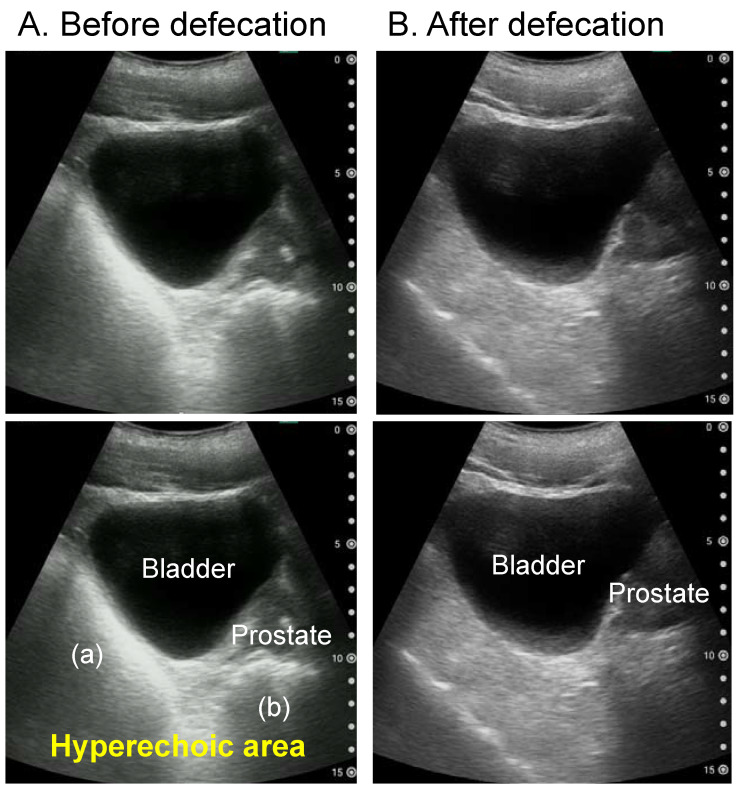
Longitudinal rectal ultrasound images of a healthy male in his 30s before (**A**) and after (**B**) defaecation. The top two images are the original ultrasound images, whereas the respective bottom images describe the findings. Before defaecation, there was faecal retention in both the upper (**a**) and lower (**b**) rectum. There is no faecal retention in rectum.

The longitudinal US image can show the location and amount of rectal faecal retention. For example, the longitudinal image can be used to determine whether the faecal retention is in the upper or lower rectum. Figure 4 shows the longitudinal rectal US images of a healthy adult before and after defaecation. There is faecal retention in both the upper and lower rectums before defaecation, whereas there is no faecal retention in either rectum after defaecation.

### 3.4. Findings That Need to Be Differentiated: Paradoxical Diarrhoea

**Figure 5:**
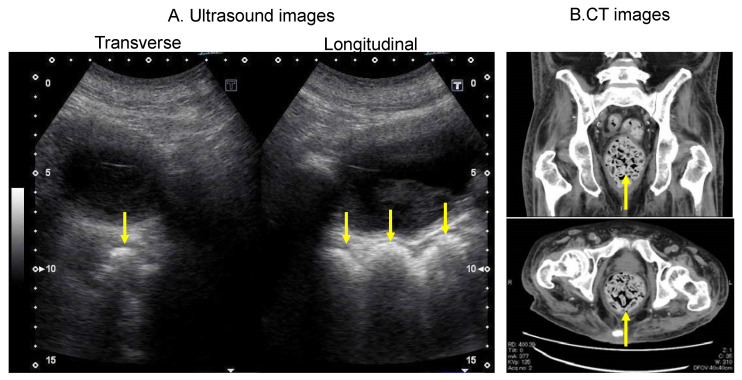
Ultrasound images of a patient who had frequent episodes of diarrhoea with intense faecal impaction in the rectum on CT imaging. (**A**) Rectal ultrasound images showing a hyperechoic area indicating faecal retention (arrows). (**B**) CT image showing faecal retention in the rectum (arrows). The upper image shows a longitudinal section, and the lower image shows a transverse section. CT, computed tomography.

Paradoxical diarrhoea needs to be differentiated in US findings [23,24]. Faecal impaction is defined as a large amount of compressed faecal retention at the level of the bowel that cannot be expelled spontaneously, causes rectal distention and results in continuous contact between the faeces and the wall, which may cause mucous membrane irritation and a resulting increase in mucous secretion [25]. In addition, faecal impaction can cause the prolonged relaxation of the internal anal sphincter; in combination with the neuropathy component of the pudendal nerve [26], this prolonged relaxation can cause watery stools to seep out around the hard mass of faeces [27]. Paradoxical diarrhoea may not be diagnosed as constipation, because watery stools are expelled gradually. Figure 5 shows a clinical image of a patient who had frequent episodes of diarrhoea, but the computed tomography image shows intense faecal impaction in the rectum. In patients with paradoxical diarrhoea, a crescent-shaped hyperechoic area with acoustic shadows can be observed.

### 3.5. Cases That Are Difficult to Observe

**Figure 6:**
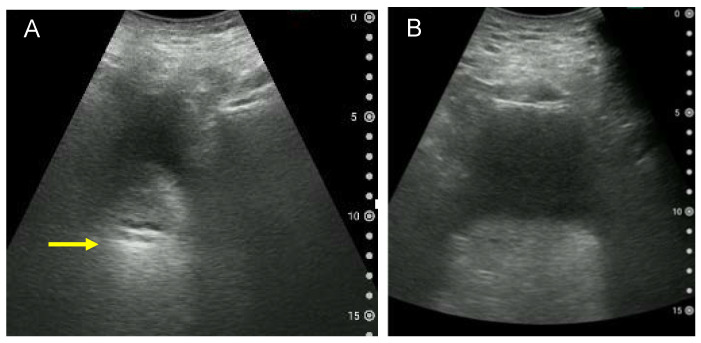
Examples of a blurred ultrasound image of the bladder and rectum. (**A**) Blurred transverse ultrasound image of the rectum due to little urine in the bladder and intestinal gas. A hyperechoic area is observed, possibly indicating faecal retention (arrow). (**B**) Transverse ultrasound image of the rectum in an adult male with a body mass index of 28.6 kg/m^2^. Due to the thickness of the abdominal wall, the bladder and rectum cannot be clearly observed. The volume of urine voided immediately after imaging is approximately 180 mL.

Obesity, the lack of urine in the bladder and gastrointestinal gas are factors that make US observation difficult. It is believed that the increased thickness of the abdominal wall due to obesity causes attenuation and scattering of the US beam, affecting the image quality when viewing the bladder and deep tissue [28]. Figure 6 shows examples of a blurred US image of the bladder and rectum. Blurred US images also occur, when there is little urine in the bladder. Therefore, whenever possible, US should be performed with at least 100 mL of urine stored. In addition, it has been reported that abdominal gas increases after eating [29]. Since gas can affect the quality of US images, it is necessary to avoid examinations just after eating or to avoid gas entering the US screen by having patients lie on their left side.

**Figure 7:**
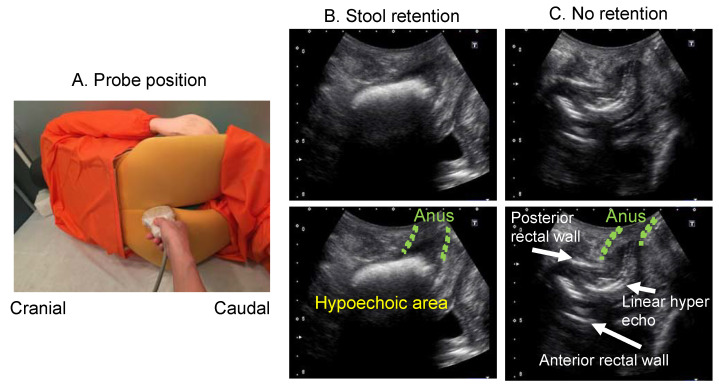
Probe position and rectal ultrasound images in intergluteal cleft scanning method. (**A**) Probe position. By placing the subject in a side-lying position and flexing the knees, a space is created between the coccyx and the anus. The probe is applied to this area with a longitudinal scan. (**B**) US image when there is stool retention. The stool in the lower rectum is observed as a hyperechoic area. (**C**) Ultrasound image when there is no stool retention. When stool is not accumulated, a hyperechoic line is observed, and even the anterior rectal wall is observed.

When observing rectal faecal retention by transabdominal US is difficult due to any of the above reasons, the intergluteal cleft approach is effective [30]. Patients should lie on their left side, with bent knees, and the medical provider should place the US probe between the patient’s tailbone and anus to confirm faecal retention in the lower rectum (Figure 7).

## 4. Future Challenges

There are several issues we need to address in the future. The first is image quality adjustment for US devices. The images presented in this article are from both portable and high-end devices, and there is no consensus on how image quality should be adjusted. The second issue is gas discrimination. As in previous studies [13,14], gas in the rectum and faecal retention were not distinguished in this study. The third issue is the development of an educational programme for US observation. It is expected that physicians and nurses who have not used US in the past will observe the rectum for diagnostic or POCUS purposes. Currently, POCUS educational programmes for nurses are being developed [16], but standardised educational materials will be necessary in the future so that multiple professions can acquire rectal US observation skills in a short time. Lastly, a system should be developed to support skills in reading US images. Reading skills are very important in the acquisition and evaluation of rectal US images. However, since reading skills are affected by differences in experience, a system that can support reading is required. A classification of rectal faecal retention findings based on deep learning has already been developed [31], and the development of US devices equipped with such a system will be necessary in the future.

This consensus document has some limitations. First, its content was not compiled through a systematic review. Just as previous studies have validated the efficacy of surgery for chronic constipation [32,33], future systematic reviews should also validate the efficacy of US assessment for constipation. Second, our consensus does not quantify the percentage of consensus among the experts, unlike what was performed in a previous study [34]. Nevertheless, this expert consensus document can help physicians, nurses and other professionals unfamiliar with US to practice POCUS for constipation, especially in the assessment of rectal feacal retention. Alongside physical examination and rectal examination, the use of US to observe faecal retention in the colon can be included in the management algorithm for identifying chronic constipation [35].

## 5. Conclusions

An expert consensus has been reached on the use of US by multidisciplinary teams to observe rectal faecal retention, evaluate constipation and select appropriate treatment and care. This consensus statement outlined a standard procedure for using US to observe the presence, properties, location and volume of rectal faecal retention.
